# Community engagement in genetics and genomics research: a qualitative study of the perspectives of genetics and genomics researchers in Uganda

**DOI:** 10.1186/s12910-023-00995-w

**Published:** 2024-01-02

**Authors:** Harriet Nankya, Edward Wamala, Vincent Pius Alibu, John Barugahare

**Affiliations:** 1 Department of Philosophy, College of Humanities and Social Sciences, Makerere University, P. O Box 7062, Kampala, Uganda; 2 Department of Biochemistry and Sports Science, College of Natural Sciences, Makerere University, P. O Box 7062, Kampala, Uganda

**Keywords:** Community Engagement, Experiences, Genetics and genomics research, Perspectives, Researchers, Uganda

## Abstract

**Background:**

Generally, there is unanimity about the value of community engagement in health-related research. There is also a growing tendency to view genetics and genomics research (GGR) as a special category of research, the conduct of which including community engagement (CE) as needing additional caution. One of the motivations of this study was to establish how differently if at all, we should think about CE in GGR.

**Aim:**

To assess the perspectives of genetics and genomics researchers in Uganda on CE in GGR.

**Method:**

A cross-sectional qualitative study was conducted at Makerere University and Uganda Virus Research Institute. Twenty-five individuals participated, the majority being male (sixteen). Participants included nineteen genetics and genomics researchers (researchers and research coordinators), two CE officers, three nurses and one nursing counsellor. Data were collected using in-depth interviews and analyzed in a thematic manner using NVivo version 12 Plus.

**Study findings:**

Thirteen of the respondents had conducted CE in their GGR in either a geographical and disease-specific community. Some respondents said CE principles are the same and there is no need for special consideration for CE in GGR. Others gave ethical issues in GGR that require special consideration for CE in such research and these were categorized into six themes: GGR is new to communities, Difficulty in communicating GGR by the researchers, Genes are shared in communities, Cultural sensitivities against GGR, Community attitude toward GGR, Some GGR studies take long to end, and Negotiation of research benefits. Special considerations for CE when conducting GGR were suggested and categorized into seven themes: creating awareness of GGR in communities, obtaining both community acceptance and individual consent, CE team composition, involve communities in solving GGR challenges, prolong CE in some GGR, develop guidelines for CE in GGR, and legal considerations on GGR.

**Conclusion:**

GGR was characterized by special issues that require special CE considerations for such research.

**Supplementary Information:**

The online version contains supplementary material available at 10.1186/s12910-023-00995-w.

## Background

Genetics and genomics research (GGR) presents enormous potential for health benefits through aiding disease management by providing insight into the host’s genetic factors that influence susceptibility to disease, disease progression, tolerance, resistance and treatment outcome [1, 2]. These benefits, however, become ethically suspect unless the process of their scientific discovery is responsive to the interests and values of individuals and communities participating in GGR. The practice of community engagement (CE) has been highly recommended internationally [3–5] and in some cases required by countries such as Uganda [6] as the best practice and strategy through which community values and perspectives can be identified and taken into consideration in research. While no universal definition for CE exists, Centers for Disease Control and Prevention (CDC) describes it as the process of working collaboratively with and through groups of people affiliated by geographic proximity, special interest, or similar situations to address issues affecting the wellbeing of those people [7, 8]. Much as the specific goals of CE may differ from one project to another, the process of CE is generally believed to, promote communities’ agency [9] enable effective access to the target community, manage health risk and community expectations and facilitate consent process [10–12]. CE is also important in building trust between the research team and participants, gathering information on the needs and expectations of the community with respect to the project and present the community with an opportunity to gain more information on the goals of the research [10]. Consequently, CE is one of the important components of the ethical research process [13, 14] in which research can be conducted in a way that is relevant to communities’ needs and priorities [15–17] that facilitates respect for communities’ ethical norms and values [15, 18].

Researchers globally have recognized the importance of CE in GGR to enable effective explanation of GGR concepts, consequently facilitating the informed consent process. CE also facilitates the return of GGR results to study participants, enables research teams again acceptance to the community and is a platform for negotiations and solving concerns involved in GGR, among other things [19–24].

Whereas much evidence has been generated about the justification, concerns, and processes of CE in research generally, the sensitivity of people’s genes suggests that there could be additional concerns that need to be addressed in GGR studies. For example, phylogenetic studies, genetic analyses of susceptibility of certain diseases such cancers, mental illness, studies around communities’ ancestry, and risks of revealing misattributed paternity intuitively suggested that there could be a need for additional caution and requirements for CE in GGR. However, limited effort has thus far been made to develop an ethical strategy that addresses community engagement in all aspects of genetics and genomic research [25]. Although some efforts have been made, especially in developing guidelines for CE in GGR [26], a number of these are international and may not guide some aspects in the local context. In Uganda, the National Council for Science and Technology (UNCST) developed the national guidelines for CE in research, but these are general and not specific to CE in GGR.

It is usually anticipated that, if researchers can assess the views of relevant stakeholders in a research process, this can allow greater understanding of the process and aid in the designing and implementation of efficient and effective practices, informed by the people that will use them [24]. Since CE requires the involvement of different stakeholders [21] including researchers, it was found necessary to elicit their perspectives on how CE in GGR should be considered. Therefore, the purpose of this study was to assess the perspectives of genetics and genomics (GG) researchers in Uganda on CE in GGR. This was to establish whether in the context of GGR there is a need for a significant shift from the way we think and go about CE in general.

## Methods

This study aimed to assess the experiences and perspectives of genetics and genomics (GG) researchers in Uganda on CE in GGR.

### Study design

This was a cross-sectional qualitative study. This design was deemed the most appropriate because this study aimed to gain a deeper understanding of the experiences and perceptions of CE in GGR and how those insights could inform whether CE for GGR should require special considerations.

### Study sites

The study sites were Makerere University and the Uganda Virus Research Institute (UVRI). These institutions were selected because of their familiarity in the conduct of GGR.

### Study team

Data were collected from April to December 2022 by a team of two: the principal investigator, who is a PhD student in applied ethics and has a Masters of Health sciences in bioethics and a Bachelor of science majoring in biochemistry. The second was a research assistant who had a Masters of Health Sciences in Bioethics and a Bachelor of Social Science with a focus in psychology. The team members had experience in conducting qualitative research.

### Data collection tool

An in-depth interview (IDI) guide was developed by the authors of this paper based on the aim of the study, scholarly views, and official guidance on good practices in CE in research and in GGR. It was then subjected to review at two separate doctoral research seminars that suggested fundamental changes and later subjected to pretesting among genomics and genetics researchers (these did not take part in the study). The research ethics committee also suggested minor edits to the guide. Generally, this process helped introduce new themes in the guide and refine the phrasing of questions. During the interviews, some adjustments were made in the guide informed by the participants’ responses.

The major questions in data collection related to; the nature of the GGR conducted, whether CE was conducted for the GGR, any special ethical issues in regards to CE in GGR, any special considerations for CE in GGR.

### Sample size

Twenty-five individuals participated in this study. The sample size was determined at the point of saturation, that is, a point at which no new ideas were being generated from additional interviews.

### Sampling procedure

The snowball sampling technique was used to identify particular GGR projects from each institution. Purposive sampling was used to select the individual participants based on their role and experience in GGR conduct. The roles under consideration were genetics/genomics researcher, research coordinator, a member involved in sensitizing and consenting participants or in charge of planning and overseeing CE in the study.

### Data collection procedure

The potential participants were approached either in person, by telephone or via email, briefed about the study and requested for a convenient appointment to participate in the IDI. Interviews were conducted physically at participants’ official working addresses, and the interviews lasted between 40 and 90 min. All interviews were conducted in English. Interviews were audio recorded after notifying the participants and obtaining their consent. Notes taking was also done by the research assistant as back-up for the interviews and for coding the identities of study participants. Participants were added up to the point at which no new ideas were being generated from additional interviews.

### Data analysis

All audio recordings from 25 IDIs were transcribed verbatim for subsequent analysis coupled with notes taken during the interview process. An inductive approach was used to code data by two coders (AT and LM). Six transcripts were manually reviewed and coded to generate the initial set of codes. This was followed by a detailed thematic analysis to develop a draft-coding framework. All transcripts were imported into NVivo version 12 Plus for coding using a framework that was iteratively revised. Using a developed coding framework, each coder performed open coding on transcripts independently, compared and assigned to relevant segments in the text. There was flexibility to accommodate emergent new themes as coding evolved. There were no major discrepancies that emerged in the coding by the two coders. However, the second coder gave detailed themes, subthemes and codes compared to the first coder. To ensure credibility of the analysis, transcripts were coded independently, compared and discussed. The revised codes were grouped into categories, and themes were identified. Emerging findings from the analysis were further discussed among NH, AT and other coauthors to strengthen the credibility of the study findings. Illustrative quotations for each emergent theme were selected for results narration.

### Ethical considerations

Ethical approval for this study was sought from the Makerere University School of Biomedical Sciences Higher Degrees Research and Ethics Committee, Ref No: SBS-2021-66. This was followed by obtaining ethical clearance from the Uganda National Council for Science and Technology (UNCST), Ref No: SS1172ES Participation in the study was voluntary, and participants’ written informed consent was sought. Serial codes instead of participants’ names were used to label participants to ensure that no identifiable information was directly linked to participants or their affiliations. COVID-19 Standard Operating Procedures as set by the Ugandan Ministry of Health were followed.

## Results

Most (16/25) of the participants were male with a mean age of 43 years (range 33–63 years). The majority (24/25) had at least a master’s degree, (15/25) were genetic/genomics researchers, and (17/25) had at least five years of experience in GGR, as summarized in Table 1.

**Table 1:** Participant characteristics

Attribute	No. of participants N = 25	Male 16	Female 9
Age			
30–39	10	5	5
40–49	9	6	3
50–59	5	5	0
60–69	1	0	1
Highest level of education attained			
Bachelors	1	0	1
Masters	13	6	7
PhD	11	10	1
Genomics research position			
Genetics/genomics researcher	15	12	3
Research coordinator	4	2	2
Community/public engagement officer	2	1	1
Research/study nurse	3	1	2
Study counsellor	1	0	1
Duration of work in GGR			
1month-4 years	8	4	4
5–10 years	10	6	4
11–15 years	4	4	0
16–20 years	1	1	0
20–25 years	2	1	1

This table is to be inserted in the results section just after the first paragraph summarizing the participants’ characteristics

Three themes emerged from the data: whether CE was conducted for GGR, whether GGR requires special CE considerations, and special CE considerations for GGR.

### Whether CE was conducted for GGR

Thirteen of the respondents had conducted CE in their GGR. One of the reasons given for engaging the community were because genes are shared in communities indicating the need to engage the whole community. Other reasons given for conducting CE were because research funders and the research ethics committees required it.

*“communities share genes and so one finding will have implications on the entire community. Therefore, genomics demands a lot of community engagement probably more than any other thing*” (IDI-12).

Some studies were reported not to require CE because they did not require a direct interface with the communities. This was reported for research in which stored samples were used and for research that neither directly impacted treatment nor had the potential to inform policy.

“*Of course, community engagement may not be feasible for certain studies; now if I get specimens and store them for future research I may have research where I am not interfacing with the people who consented to give the samples and even the results that are going to come back are what we call basic science studies. If it does not have a direct impact on policy, it doesn’t have a direct impact on treatment of these patients and so on, of course there is no need to have community engagement because it is not truly directly interfacing with the communities.”* (IDI-5).

Others reported not to have conducted CE because their research was too short to have a continuous relationship with the community/participants.

*“CE depends on the type of study unless it is a ten year or five-year longitudinal kind of study that is continuous. You’re not going to tell someone who has studied for one year to keep engaging in what?”* (IDI-6).

#### Types of communities engaged

The communities that had been engaged included; those affiliated by geographic proximity and those at high risk of target genetic diseases/conditions like TB, HIV, trypanosomiasis, schistosomiasis, cancer, and psychiatric disorders.

#### Extent of engagement

A few respondents reported to have engaged their communities at the conception of the research. Most respondents engaged their communities at sample collection. They approached the community after developing the proposal and then explained to them what the research was about, the objectives, how it was to be conducted, who was eligible, who they were targeting, their anticipations, the risks and benefits.

*“Therefore, the time that I engaged with the participants was during the time of consenting and enrollment, and because we did not see the need of returning results, so we only saw the patients once, at the time of enrollment. In addition, then after that, we did not see them again, we did not follow them.”* (IDI-15).

None of the respondents in this study had returned GGR results to their participants or their communities, much as return of results was reported as the main expectation communities had. In their explanation, it takes long at times to get results because some samples have to be shipped abroad for further analysis or samples have to be compared with samples from other sites, which delays the process. Others did not just consider returning because the results could not provide clinical value to the participants.

*“The truth is that ever since we started doing these kinds of genomic studies, we haven’t issued any results, it’s now eight years, we haven’t given out any results.”* (IDI 21).

*“Many times, actually the community is interested in receiving their results at the end of the day, that one they want to know, that is a major expectation from the community.”* (IDI-12).

### Whether GGR requires special CE considerations

When asked if CE in GGR should be handled in a special way, some respondents said no; that the principles of CE were guiding enough for all research even GGR.

*Community engagement is plain community engagement; find the relevance of what you’re doing, how is it relevant in the population that’s how you’re going to engage that’s it like for any kind of study*. (IDI-6)

*The way our stakeholder engagement is structured is known internationally whether it is genomics. For whatever use, there are those tendencies that are at the top then you go down to now saying for me am looking at genomics but the truth of engagement is engagement, so, whether you try to add the title genomics, it is engagement. Therefore, it has certain principles that cut across which you cannot say that because am doing genomics, am not going to talk about it*, no. (IDI-8)

However, some respondents gave particular ethical aspects in GGR that call for special measures when engaging communities in such research. These were; Genes are shared in communities, GGR is new to communities, Difficulty in communicating GGR by the researchers, Cultural sensitivities against GGR, Community attitude toward GGR, Some GGR studies take long to end, and Negotiation of research benefits.

#### Genes are shared in communities

Respondents said genes are in some cases shared by members in communities affiliated by geographic proximity and hence knowing the genes of one individual has high chances of knowing about the individual’s family or community. They indicated that for this reason, there is a stronger need to engage whole communities.

*“Communities still share genes and so one finding will have implications on the entire community. Therefore, genomics demands a lot of community engagement probably more than any other thing*” (IDI-12).

Respondents also said that since genes are shared in communities, genetic findings have social implications and cited of these was the possibility for family conflicts arising from DNA paternity confirmation.

*“Researchers need to understand there is individual risk while there is collective risk that it is most apparent in genetics research where there is an invisible network of interconnectedness so that what you learn here will tell you many things about others” (IDI-25)*.

*“The other issue has to do with the whole identity question and it has been very common, those issues of paternity and family life and when you do the DNA testing and then you discover the particular child is not actually part of this family in question, what do you do? Those are some things that are major ethical issues in genomics research that must be taken care of”* (IDI-23).

Respondents mentioned that because of shared genes, there rises conflicts the participant’s right and the community’s rights especially on issues of who to consent for the community genes, who to return the results to, and who shares the research benefits.

*“If the participant does not agree saying; ‘it’s only me to know something about me’, what happens to the community? The community also might demand to know the results; “do we have such and such a problem in the community?”* (IDI-12).

Some respondents suggested that researchers need to identify who has the responsibility to consent for the shared genes in the community because failure to do so was said to lead to rejection of the study.

*“There is a time somebody was to do a study on mitochondria of baganda and the question was that should the Kabaka (king) consent? Because who consents on behalf of the Baganda?… Therefore, the study was rejected? Yes”* (IDI-12).

#### GGR is new to communities

The main reason respondents gave for engaging communities in GGR was because GG was still new to communities and to Africa at large, so there was a need to create awareness so people get to know and contribute to it.

*“First, this is still new, the genomic and genetic studies is very new. The other time I gave you statistics that Africa represents 1% contribution to genetic studies and yet to be a big part of the world, so, that tells you that genomic studies are new. Therefore, if you have something new, it is a responsibility that you truly need to engage the community more so that they are aware.”* (IDI-12).

Respondents said that because of the communities’ low understanding of GGR, researchers underestimate the communities’ contribution to the research on GG science.

#### Difficulty in communicating GGR by the researchers

Explanation of a number of GGR terminologies, concepts and procedures was reported to be difficult. Although some languages in Uganda had come up with equivalent words for some terminologies, such as DNA and gene, equivalents to terminologies such as mutation and more were said to still be challenging.

*“Many of these things, if you are talking in the local language, there is no appropriate language to communicate. Maybe when you are talking of DNA, Gene, but when you go to mutation, and then say susceptibility.*.” (IDI-12).

Some GGR procedures were also said to require special explanation to the communities. Listed among these were the collection of large samples, sample storage and shipping.

*“They must clearly understand why you’re storing the samples, how long you’re storing the samples? Are you going to ship the samples for other collaborators at some point! These are things that come up when you take a sample from someone, there are so many questions that could come up, especially in Africa, blood is essential”.* (IDI-9).

How to explain the meaning of GGR results to participants in a way that they understand was reported a challenge. They said that explaining susceptibility to disease or to a genetic condition in a sense that it is just a possibility to the condition, without causing worry was a challenge.

*“how to explain to participants the meaning of their results in a way that they will truly understand would be a challenge. How do I explain to those who are at higher risk of getting a genetic condition? It would be a challenge explaining a condition if it does not lead to disease, that this, is not a cause of disease, but it is a risk factor. How do you truly explain it to this person in a way that one, they will not be worried about their lives in a way that they will understand that they’re just at risk. In addition, maybe they just have to maybe change their behavior, I would see a challenge there”.* (IDI-4)

Additionally, respondents said that some genomic studies do not involve clear-cut genes; it is a mixture or actually an interaction between the genes and many things, such as environmental factors, which makes it tricky to explain their actuality of occurrence.

*“Ours are kind of tricky when we go to psychiatric research, there are no key cut genes, it is a mixture or actually an interaction between the genes and so many things like environmental factors, so it becomes tricky because we are trying to determine the patterns that are involved*.” (IDI-1).

#### Cultural sensitivities against GGR

Respondents suggested that GGR researchers need to identify cultural and religious beliefs, norms and practices in their communities. They said that some religions are against genetic procedures such as gene modification. Respondents also reported cultural beliefs attached to GGR including; conspiracy theories, myths and misconceptions such as the association of genetic disorders with witchcraft, hesitance to give samples in fear of being used for witchcraft, and concerns with sample storage.

*“If you find those who are basically grounded in some religion, sometimes you feel it’s not right for you to talk about certain things and genetic modification”*. (IDI-8)

*“People are always worried why we are picking their samples, people ask what are you going to use this information for, are you going to use it for witchcraft, something like that”.* (IDI-20)

Respondents reported some communities to have cultural practices that were contrary to conventional medicine and DNA testing. These included testing for paternity by resemblance.

*“The identification of paternity is based on whom you resemble and yet this can be explained by genomics by comparing DNA from father to the grandfather.”* (IDI-20).

#### Community attitude toward GGR

Respondents said that some communities have attitudes toward GGR that may hinder them from accepting the research. Communities were reported to be concerned that their DNA would be used against them, that is, to wipe them out.

*“However, of course there are those many perceptions of why you are looking at my DNA, are you using this to develop drugs to wipe us out, are you trying to track us, are you trying to do this so there are those kinds of perceptions that may come up”.* (IDI 5)

Other respondents reported an indifference of community members on how their genetic samples would be used as long as they received compensation for participating in the research.

*“Genomics research is not having any problems in Africa, is there anything we have failed to do because of our cultural norms, like what? In fact, if you can do anything genomic, it is the best place because you can get any sample you want. People here as long as you talk to them nicely, compensate them… We have started a sperm bank, people are coming they get compensated they give us their sperm so here is the best place to do your research”.* (IDI-6)

Communities were said to mainly consider GGR in terms of genetic/DNA testing. They normally anticipate the research to enable them to confirm paternity, which has made some people hesitant to accept the research. Paternity was said to be feared for causing conflicts and family break-ups.

*“When they hear about genes, the first thing that drops down their minds to some is DNA… they are like okay doctor I thought you wanted to check if I’m the biological parent so after you explain to them they are okay”.* (IDI-14)

Respondents also mentioned that the community’s attitude towards GGR was prune to change after them acquiring knowledge on genetics and genomics.

*“In making them aware, if people have understood very well, you expect some challenge like; I’m not ready for that”* (IDI-12).

Respondents reported that communities had diagnostic and therapeutic misconceptions about the intensions of the research. Communities were reported to expect to obtain healthcare from GGR; that is, they hoped to receive treatment and prevention for their genetic conditions. Others expected financial benefits from the research.

#### Some GGR studies take long to end

GGR was reported to have the possibility of delaying to return GGR results. The delay was attributed to the time and resources required to derive conclusive results.

*“The truth is that ever since we started doing these kinds of genomic studies, we haven’t issued any results, it’s now eight years, we haven’t given out any results.”* (IDI 21).

*“by the nature of genomics before you truly come out conclusive and say this is the issue, or this is the case, there are many steps for example, sometimes the technology is not local or the technology will be far but even abroad there could be a que. However, that data must be analyzed so it takes time”*. (IDI-12)

Delayed results were said to lead to research fatigue by participants, resulting in hesitance to keep participating in the research. Researchers also reported difficulty in keeping a working relationship with study participants after data collection. They said during the time of working on the results, they may not have valuable information to discuss with the community.

*“I think it is very difficult (to keep a working relationship) for example I go to the village here, collect my sample, and am busy working out and all those things so for five or ten years I may not have any reason to go back. Therefore, it is hard to just go and say hi people, how are you? So, at least you have to have something to share with them”* (IDI-2).

#### Negotiation of research benefits

Respondents reported challenges of how to handle research benefits between the participants that provide the genetic sample, the communities that share that gene and the GG researchers. The debatable benefit reported was intellectual property.

*“If whatever you discover has commercial, intellectual property, how do you handle the patient, how do you handle the community, how do you benefit as the researcher?”* (IDI-12).

The other GGR benefit concern was how possible the research community will access research outputs, such as gene therapy, since it will not be affordable to the communities.

*“Are you able to provide to the study population like ethically it would demand if there is a known remedy, the study participant should have benefit from that. However, now, if you find it, and they cannot afford it? …like gene therapy for sickle cell anemia, but can they afford it?”* (IDI-12).

### Special CE considerations for GGR

When asked for the special CE considerations for GGR, respondents suggested creating awareness for GGR in communities, obtaining both community acceptance and individual consent, special CE team composition, involving communities in solving GGR challenges, developing guidelines for CE in GGR, and legal considerations for GGR.

#### Creating awareness for GGR in communities

Respondents opined the training of communities in genetics and genomics to enlighten them more on its scientific relevance. This was said to be done through continuous communication to communities to allow them get to hear the same message over time, so they appreciate the research.

*“It is good that stakeholders actually learn of what happens in genomics research and what could be the usefulness of genomics work for the community”.* (IDI-6)

“*Discuss with them and if you think they are refusing for either ignorance or lack of information continuously engage them, convince church leaders, get opinion leaders engage to believe and know your research is important short of that you can’t do research” (IDI-24).*

In ensuring that the community understands, respondents suggested ensuring that the community leaders understand the genetics and genomics first such that they can explain it to their community members. Respondents also highlighted that researchers should use a consultative approach to communicating with the community. They said that with this, they learn about the cultural sensitivities, concerns and what the community knows in relation to GGR.

*“Respect and approach them in a way of you seeking to share knowledge not that you are giving them knowledge and not what we call instructions, looking for information from them to share with you, then to share with them what you know. Identify community concerns, their cultural sensitivities”* (IDI-17).

#### Obtain both community acceptance and individual consent

Respondents suggested that the community should accept the research first for the researcher to be able to access the participants to seek their individual consent.

*“Therefore, if the community thinks this is not a good thing, then you have a problem truly moving on with the consent”.* IDI-12.

Regarding the study findings, respondents emphasized that the researchers should consent to the participants first before revealing the findings to their families and communities.

*“This participant can consent how to or give information on how to handle it. This is because you’re protecting the privacy of the participant yet you know it is going to affect his/her family”.* (IDI-10).

#### The CE team composition

Respondents opined to researchers having a CE team that is culturally and professionally diverse. For cultural diversity, the team should include individuals that understand the culture where the research is intended to be conducted. This was said to enable easy communication and earn trust from the communities. Professional members were said to include genetic counsellors to explaining the research procedures, and to help the community and participants in handling the psychosocial effects of the research and in, return of GGR results and offer psychosocial support to.

*“I think it will also need to have a genetic counsellor because there are potential relationship issues there. Therefore, someone might need either a counsellor or psychologist, someone to help them deal with the emotional and mental issues that can arise”.* (IDI-16).

Respondents also recommended research teams to set up Community Advisory Boards (CAB) and train them on how to mobilize the community for genomics research. CABs were also thought to be links between the researchers and the community to provide insights and communicate to the researchers any issues involved in the research.

*“To have a community advisory team or group, it may be of five people it could include maybe a religious leader, community leader or a cultural person such that they are in touch with the community”* (IDI-22).

Respondents opined that the CE team should be trained on genomics science and on how to plan and implement CE by emphasizing the need to base on the possible risks to the community, the benefits and the anticipated results and their implications.

*“For the researchers that are going to give the information to the community, you have to first be sure they have the clear explanation or you first train them so that they get better information about genomics*” (IDI-10).

#### Involve communities in solving GGR challenges

Communities should be part of problem solving for GGR; they help in forming translations to GG terminologies and in allaying anxieties.

*“Communities are part of the solution that they have now come up with new names of DNA, they have new names of RNA”.* (IDI-12)

*“And when you are disseminating results, sometimes communities will also help first of all allay their anxiety”* (IDI-12).

#### Prolonged CE in GGR

For delay in return of GGR results, respondents recommended that the CE should be prolonged through researchers continuously updating the community on the progress of the research, as one respondent stated:

*“I think maybe continually communicating with them. Reminding them, just do not go silent. Maybe keep updating them that; “you know, the results when they’re out, we will communicate them this way”.* (IDI-15)

Some respondents suggested the feeding back of preliminary findings to communities as researchers work at validating the GGR results.

*“You can actually share the glucose levels when you know that one is right so you share it down then tell them the final results of this genomics study will take some time because we need to give validated results so you can just give them something to allow them continue*.” (IDI-12).

Even after results are returned, genomics researchers were advised to maintain a good relationship through advising participants to go for testing if predisposed to a genetic disease.

*“Therefore, you could actually go beyond like, you could say that this is what we found or maybe you can advise them to go and test for that thing. You may actually go beyond, depending on the scope of the research”* (IDI-2).

#### Develop guidelines for CE in GGR

Respondents pointed to the need for guidelines specifying how CE for GGR should be done.

*“Can we have guidelines on how to do that (community engagement) because I can’t determine how I can take this one, so it is necessary to have guidelines on how to go about it”.* (IDI 1)

A few respondents knew of some international guidelines for CE in GGR, for example, those developed by the Human Heredity and Health in Africa (H3Africa). A few were still aware of the ‘National guidelines for protection of Humans as Research participants’ that were under revision and would potentially have a section of GGR. Additionally, hardly any of them knew about the developed ‘National guidelines for CE in research’ that had been launched recently.

#### Legal considerations for GGR

Respondents also advocated for stringent legal requirements for GGR, especially regarding genomic data and results handling, since these were thought to have the potential to discriminate among people on several grounds, such as race.

*“The requirements should be more stringent. Why? Because that can even be used to generate results that could lead to stigmatization, discrimination with regards to race with tribes and all that stuff…So what I’m trying to say is that if it pertains to human genomic data, requirements should be very stringent. In addition, it becomes even more sensitive”* (IDI-9).

## Discussion

GGR is still new to Ugandan communities, and for them to be able to contribute to such research requires their understanding of it. As reported in this study, researchers did not find it necessary to involve communities at the inception of the research because they thought communities would have no valuable contribution to the research. Actually, not all scientists value lay contributions and many community members feel insecure about the importance of their contributions [18, 30]. This reason for not engaging communities at the conception of GGR is the same reason why researchers should be required to do so. GGR researchers should be made to bear much stronger obligations to ensure that the communities and research participants gain sufficient comprehension of the study and the various potential benefits and risks it presents to be able to make informed decisions in regards to that research. This is important, especially considering that, as revealed by one of the respondents, sometimes when potential participants gain significant understanding of the risks involved in GGR, they decide not to participate.

Genes are shared among members in a family and even the community. Given that CE is aimed at involving parties affected by the intended research [31, 32], GGR, therefore, requires extended engagement beyond the participants to the entire. The community has to be part and advise on what decisions to take in regards to the research for example; who makes the decisions on behalf of the community, and how the study benefits are to be shared, among other things. However, much as the community has a right in the study proceedings, the participants’ rights have to still be accorded by the researcher [33, 34].

Whatever is known about an individual’s genes can have implications for the rest of the people who share that gene. This would have dire social consequences for the community, especially if a statistically significant number of samples are collected from individuals in a generically related group, analyzed and stigmatizing results conclusively obtained. With as many as 65 indigenous communities in Uganda [35], which most people believe to bear close genetic relations, and the already existing stereotypes against each of these, the heightened social concerns about GGR and its regulation become stronger.

CE in GGR stands to take longer than for other kinds of research because it may involve following up a gene-specific community that may be scattered; GGR’s potential for prolonged future research on stored samples; and as reported in this study, the potential for some GGR results to take long to be returned. Respondents should maintain lasting relationships with the communities because, as reported in this study, communities experience research fatigue while waiting, which results in hesitance to continue engaging in the research. Additionally, the potential psychosocial implications from findings would require researchers to maintain contact with the communities and provide them support. However, respondents reported finding it a challenge to maintain these lasting relationships because they lack valuable information to share with the communities at the time lag between sample collection and return of results. Consequently, questions arise on how long the engagement should be extended after the study has ended and, in case of delayed results, for how long the community should keep waiting.

Communities were reported to have cultural practices that mimic some genetic procedures, and the most frequently mentioned was paternity testing. This indicates the possibility of such communities refuting GGR on grounds of research imperialism, which threatens to replace their cultural practices. Communities are also skeptical of some genetic samples, such as hair and nails, since in their cultures, such body parts can be used for witchcraft. Some of their religious ideologies also contradict GGR since they see it as a manipulation of God’s plan. These concerns have been reported even in the literature [36–38]. Such community values and concerns can be realized through CE to enable researchers to respect and address them.

The community attitudes toward GGR as highlighted in this study portrayed an ignorance that communities have on what their genes could be used for, especially in risky endeavors. Communities consider research as an avenue to gain financial benefit in the form of compensation. As stated by one of the respondents in this study, as long as they are compensated for participation, people in Africa do not care what their samples would be used for. This indicates an economic vulnerability that impedes communities’ decision-making capacity toward GGR. This points to the need to sensitize these communities on the potential risks their samples stand to be used in order to avoid being violated. However, in sensitizing communities, it was feared that communities could obtain negative attitudes toward GGR arising from learning more about the research, especially the risks that would come with it. This was also portrayed in a study by Watanabe and colleagues [39].

Communities were also said to have therapeutic misconceptions about GGR; that is, they hoped the research would enable them to know their genetic status and that of their families and obtain prevention and treatment for their genetic conditions. Research misconceptions are common even in other biomedical studies [40–42]. All these points point to the need for an engagement where all such misconceptions are identified and addressed.

In addressing the special considerations for CE in GGR, respondents suggested the creation of awareness of genetics and genomics in the communities. As a requirement for ethical decision making is the comprehension of the research. If communities’ knowledge is boosted, they will be empowered to make informed decisions about the research. As reported in this study, the continuous sharing of information allows people to hear the message repeatedly, ask questions that get answered which improves their understanding of concepts. This continuous approach is evident in Uganda where communities are now familiar with genes and DNA testing because of the continuing DNA tests for paternity that are conducted and the reports of which aired on local mass media [43]. This has prompted some languages for example, the Baganda to find equivalent words for DNA. However, as reported in this study, rare genetic terminologies such as mutations are still hard to translate, so there is a need for more collaboration of researchers with communities to find equivalent words to such terminologies. It was suggested in this study that community leaders should be taught first so they teach their communities about the research. This shows that researchers should respect the leadership structure in communities. It also portrays the trust that communities have in their leaders, which further emphasizes the need for CE so that those hierarchies are followed. However, researchers should be cautious of the authoritarian possibility that could arise out of this priory given to leaders so it does not cloud community members’ agency.

Given that genes are shared in communities, this study highlighted that both individual rights and community rights should be respected. The community has to first accept the GGR to be conducted, and then individuals are in a position to consent and participate in it. However, beyond that, the participants’ consent comes prior to the community’s demands. It was said, for example, that to be able to share research findings to communities, the participant has to consent to that. However, because of the implications the results could have, some studies have suggested that if the results are anticipated to have implications beyond the participant, that should be explained to the community prior to study recruitment and guidance on that informed by the community [25, 44–46].

It was suggested that the CE team for GGR should have a genetic counsellor given the psychosocial implications of this kind of research. The requirement for genetic counsellors has been emphasized much in studies on people’s genes [47–49]. However, in Uganda, as in a majority of African countries, there are no qualified genetic counsellors [50, 51]. This calls for a need to train for such positions since their absence could affect the uptake of GGR in communities in fear of the psychosocial issues involved. Genetic counsellors are expected to be better positioned to prepare the communities for the research and how to handle the potential psychosocial implications involved.

Given the reported prolonged CE for GGR, there is a need for a higher CE budget for the research team to be in position to maintain lasting relationships with the communities. Similar to this study, a challenge in maintaining long-lasting relationships with communities was also reported in a study by Amy and colleagues [23]. Both studies thought researchers could address this challenge by engaging and establishing professional collaborations with community-based researchers or leaders from the originating communities as major stakeholders in collaborative research efforts. They thought this approach can benefit the research by leveraging the experience and knowledge of the local community leaders or researchers about their communities while increasing the probability of long-term engagement with the communities through local members of the research group.

The development of specific guidelines for CE in GGR was reported as a measure that will encompass all the ethical considerations in CE for GGR. Uganda has recently developed national guidelines for CE in research (citation). However, these are general, and as this study’s findings have indicated, there is a need for special consideration for CE in GGR. Concurrently, the Ugandan National Council for Science and Technology is revising the ‘National guidelines for protection of Humans as Research participants’ and these are expected to have a section on GGR. The findings of this study will potentially inform the revision of some of those national guidelines. In addition, the findings of this study call for the development of specific guidelines for GGR, and these should have considerations for CE, as highlighted in this study and in the literature.

### Limitations

The study reported in this manuscript involved only researchers, yet the perceptions of other key stakeholders, such as community stakeholders and research regulators, would have provided a more complete picture of the perceptions regarding CE in GGR. However, similar work involving various research stakeholders is currently ongoing by the same research team which we hope will enrich the available data.

## Conclusions

GGR requires special CE considerations given the issues involved in such research. GGR is new and thus requires community sensitization to empower communities to provide valuable input to such research. Genes are shared in communities, so the implications of the research are extended to others in the community implying the need to engage them too. CE for GGR takes longer if issues such as delays in obtaining conclusive results are considered. The cultural sensitivities and attitudes toward GGR implied thorough community consultation. Some of the special considerations for CE in GGR include; development of specific guidelines for CE in GGR, planning for a bigger CE, forming more lasting relationships with communities to cater for the extended CE in GGR, and having genetic counsellors on the CE to specially support the handling of psychosocial implications involved in GGR.

### Electronic supplementary material

Below is the link to the electronic supplementary material.

Supplementary Material 1

## Data Availability

All data generated or analyzed during this study are included in this published article.

## Abbreviations

CAB: Community Advisory Board
CE: Community engagement
GG: Genetics and genomics
GGR: Genetics and genomics research
IDI: In-depth interview
REC: Research Ethics Committee
UNCST: Uganda National Council for Science and Technology
UVRI: Uganda Virus Research Institute
