# Supplementary material for: Community engagement in genetics and genomics research: a qualitative study of the perspectives of genetics and genomics researchers in Uganda
Source: BMC Med Ethics. 2024 Jan 2;25:1. doi: 10.1186/s12910-023-00995-w (PMC10763360; doi:10.1186/s12910-023-00995-w)
Supplement: Supplementary file 1 — Supplementary Material 1 [file 12910_2023_995_MOESM1_ESM.docx]

**IN-DEPTH INTERVIEW GUIDE**

**Title: Community engagement in genetics and genomics research: a qualitative study of the perspectives of genetics and genomics researchers in Uganda.**

***Introduction:*** You have been identified to potentially participate in this study because you conducted/ are conducting genetics and/or genomics research, so you have knowledge and experience relevant to this topic of discussion.

**Background information**

I am going to ask you some questions about your background. This information helps us to know about our respondents (your identity will remain confidential)

|  | Sex (observe) | 1. Male 2. Female 3. Other |
| --- | --- | --- |
|  | Place of work/ institution | ……………………………………………………………………… |
|  | Occupation/position | ……………………………………………………………………… |
|  | Field of specialization | ……………………………………………………………………… |
|  | Highest level of education attained | 1. Bachelors 2. Masters 3. PhD   Other(specify) ……………………………………………………………………… |
|  | Duration of participation in GGR research | ……………………………………………………………………… |
|  | How old are you? | ………………… Years  Prefer not to say |

**Discussion Questions**

**Experience on CE for genetics/genomics research**

1. What was/is the nature of the genetics/genomics research that you were/are involved in?
2. How would you explain the concept of community engagement (CE)? (*If not well versed then explain the concept*)
3. In carrying out your genetics/genomics research, did/are you do/doing any CE? Why/why not? (*If not then proceed by asking in future tense*). What was/is your CE goal?
4. What was your target community? At what stage of your study did you involve the target community and why at that stage?
5. How did/ would you access your community? Which particular individuals/groups did you work with in the community and why?
6. It is usually recommended that to be effective, CE should start from conception of the study to; planning, implementation, and reporting back of findings and beyond (depending). How possible is this for genetics/ genomics research? Give a brief explanation.
7. What challenges did you encounter while engaging your community in genomics research? What recommendations would you give to solve such challenges?

1. What guidance did/would you follow when engaging communities in genetics/genomics research?

**Perspectives on CE in genomics research**

1. What ethical issues that pertain to genetics/ genomics research are you aware of?
2. CE is recognized on principle but the knowledge base on how it should be effectively conducted in genetics/genomics research is limited. What is your view on that?
3. Do you think GGR requires unique CE considerations? Why/Why not?
4. In your view what key considerations should CE in genetics/genomics have?

**Other questions**

1. Do you have any questions, additions or concerns in line with our topic of discussion?

Okay, thank you very much for your time and very useful ideas.
